# 2846. Microbiological Characteristics of the Patients with Acute Pyelonephritis who experienced Urinary Tract Infections within prior to 1 year

**DOI:** 10.1093/ofid/ofad500.2456

**Published:** 2023-11-27

**Authors:** Choseok Yoon, Jeoungyeon Kim, Wooyoung Jang, Ji Won Go, Jinnam Kim, Yangsoon Lee, Bongyoung Kim

**Affiliations:** Hanyang University Seoul Hosptial, Seoul, Seoul-t'ukpyolsi, Republic of Korea; Hanyang University Seoul Hosptial, Seoul, Seoul-t'ukpyolsi, Republic of Korea; Hanyang University College of Medicine, Seoul, Seoul-t'ukpyolsi, Republic of Korea; Hanyang University College of Medicine, Seoul, Seoul-t'ukpyolsi, Republic of Korea; Hanyang University Seoul Hosptial, Seoul, Seoul-t'ukpyolsi, Republic of Korea; Department of laboratory medicine, Hanyang University College of Medicine, Seoul, Seoul-t'ukpyolsi, Republic of Korea; Department of Internal Medicine, Hanyang University College of Medicine, Seongdong-gu, Seoul-t'ukpyolsi, Republic of Korea

## Abstract

**Background:**

Approximately 15% of patients with acute pyelonephritis (APN) experience another urinary tract infection (UTI) within 1 year in South Korea. The purpose of this study is to examine the microbiological characteristics of patients with APN who experienced UTIs within prior to 1 year.

**Methods:**

A retrospective cohort study was performed in a tertiary-care hospital in South Korea from July 2019 to December 2021. All female patients who were diagnosed with community acquired APN at admission were recruited. Patients who had prolonged hospitalization period due to medical problems that were not associated with APN treatment were excluded. We regarded recurrent group as the cases which have history of UTI within 1 year. Types of causative organism, major antibiotic resistance of *E.coli* isolated from patients' urine or blood, and the genetic characteristics of *E.coli* were compared between recurrent group and non-recurrent group.

**Results:**

A total of 285 APN patients were finally included in this study, of which 41 (14.4%) were belong to recurrent group. *E.coli* was the most common causative pathogens in both groups, but the proportions of some pathogens, such as *Klebsiella pneumonia* (17.1 vs. 4.7%, *P=0.007*) and *Pseudomonas aeruginosa* (5.7 vs. 0.5%, *P=0.014*) were higher in the recurrent group than the non-recurrent group. There were no significant differences between the two groups in the proportions of antibiotic resistances to *E.coli*, such as amikacin (0 vs. 1.2%, *P=0.597*), cefotaxime (25.0 vs. 22.5%, *P=0.788*), and fluoroquinolone (20.8 vs. 28.9%, *P=0.409*), etc. The most prevalent β-lactamase and PMQR determinant were CTX-M-1G (44.4 vs 60.0%, *P=0.308*) and *aac(6’)-lb-cr* (50.0 vs 36.2%, *P=0.303*), respectively, and the predominant clone was ST131 (22.2 vs 17.8%, *P=0.744*) in both groups. However, there were no significant differences in the proportions of phylogenic groups, resistance genes, and virulence factors between the two groups.

**Figure:**
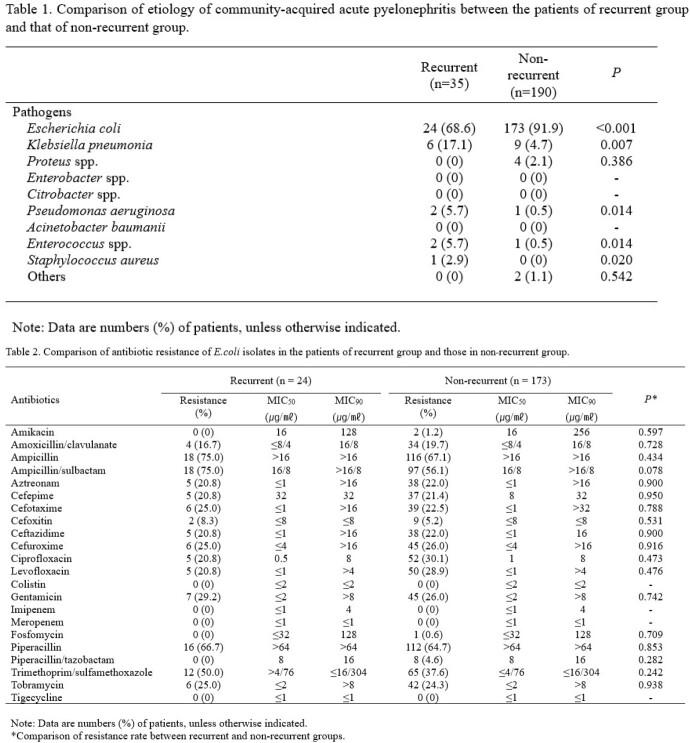

**Figure:**
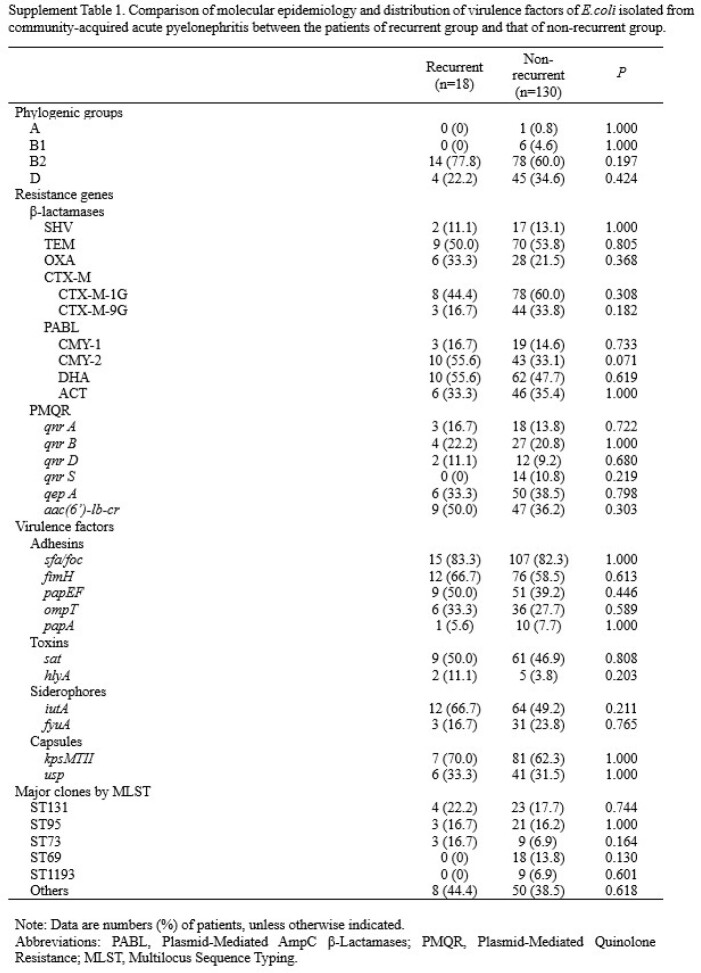

**Figure:**
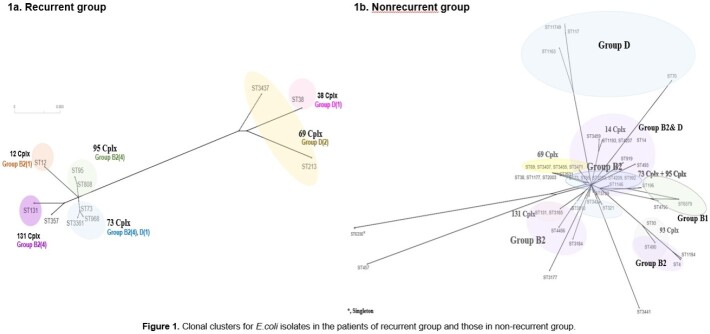

**Conclusion:**

*E.coli* was the most common causative pathogens in both groups, but the proportion of pathogens except *E.coli* was higher in the recurrent group than the non-recurrent group. There were no significant differences between the two groups in the proportions of antibiotic resistances to *E.coli*, phylogenic groups, resistance genes, and virulence factors.

**Figure:**
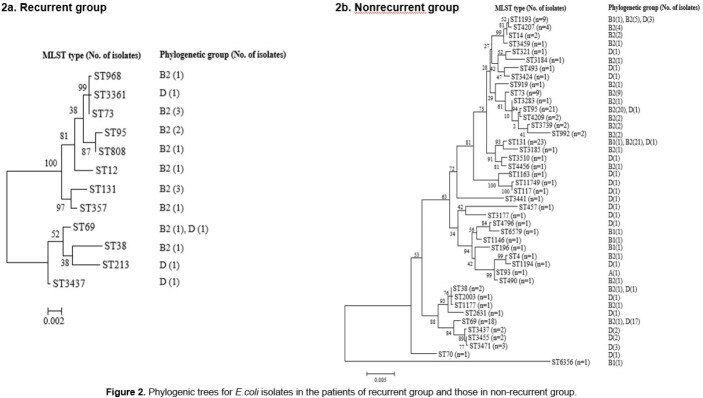

**Disclosures:**

**All Authors**: No reported disclosures

